# The preface: Nickelates—a new playground for high-*T*_c_ superconductivity

**DOI:** 10.1093/nsr/nwaf322

**Published:** 2025-08-18

**Authors:** Meng Wang, Fu-Chun Zhang

**Affiliations:** Guangdong Provincial Key Laboratory of Magnetoelectric Physics and Devices, School of Physics, Sun Yat-Sen University, China; Kavli Institute for Theoretical Sciences, University of Chinese Academy of Sciences, China

## Abstract

This editorial article introduces 5 research articles and 1 review article in NSR special topic: Uncovering the mechanism of nickelate superconductors.

The pursuit of high-temperature superconductors, characterized by a superconducting critical temperature (*T*_c_) exceeding the McMillan limit, remains a central frontier in condensed matter physics. The experimental journey into nickelate superconductivity began in 2019 with the observation of superconductivity below 15 K in doped infinite-layer nickelate Nd_0.8_Sr_0.2_NiO_2_ [1]. Subsequent discovery of superconductivity near 80 K in the bilayer Ruddlesden–Popper (RP) phase nickelate La_3_Ni_2_O_7_ under pressure spurred extensive research into the RP phases of nickelates [2]. A recent breakthrough, the realization of superconductivity above 40 K at ambient pressure in thin-film bilayer RP nickelates, further solidifies nickelates as a distinct high-*T*_c_ family alongside cuprates and iron-based superconductors [3,4]. The *NSR* Special Topic reviews the status of nickelate superconductors and presents experimental and theoretical studies probing the superconducting mechanism in both bulk and thin-film samples.

The review paper by Wang *et al.* summarizes progress since the theoretical proposal of superconductivity in doped LaNiO_2_ last century [5]. Nickelates exhibit diverse structures and valence states. While hole-doped infinite-layer ‘112’ nickelates share similarities with cuprates, possessing a 3*d*^9^ configuration and a hole-doping phase diagram, key differences emerge: their parent state is a Mott insulator rather than a charge transfer insulator, and their electronic structure involves significant contributions from rare-earth 5*d* orbitals. The review details progress and challenges in RP nickelates, including high-pressure synthesis requirements, sample quality issues (apical oxygen vacancies, stacking faults) and widely studied spin/charge density waves at ambient pressure. Discoveries of superconductivity in other pressurized RP phases (e.g. La_4_Ni_3_O_10_ and La_5_Ni_3_O_11_) and ambient-pressure thin-film La_3_Ni_2_O_7_ samples offer new pathways to elucidate the superconducting mechanism.

Direct diamagnetic susceptibility measurements under high pressure are challenging. In a research article, Li *et al.* report the first measurements of the Meissner effect in La_3_Ni_2_O_7_ using a superconducting quantum interference device (SQUID), extending the superconducting phase diagram up to 104 GPa [6]. They find superconductivity emerges coincidently with the straightening of the Ni–O–Ni bond angle along the *c*-axis. Wen *et al.* present a study directly imaging the Meissner effect in bilayer RP nickelates using nitrogen-vacancy centers on diamond anvil culets [7]. Combined with simultaneous Raman measurements, their work highlights the impact of inhomogeneous pressure on structural transitions. Wang *et al.* propose a theoretical model for bilayer RP nickelates as a self-doped molecular Mott insulator, where superconductivity may arise from two nearly degenerate antisymmetric molecular orbitals of *d*_*x*^2^-*y*^2^_ and *d*_*z*^2^_ [8].

Leveraging the gigantic oxidative atomically layer-by-layer epitaxy method, Li *et al.* grew high-quality superconducting La_2.85_Pr_0.15_Ni_2_O_7_ thin films with *T*_c_ above 40 K on SrLaAlO_4_ substrates, enabling band structure measurements in the superconducting state [9]. Their angle-resolved photoemission spectroscopy (ARPES) study reveals contributions from both Ni *d*_*x*^2^-*y*^2^_ and *d*_*z*^2^_ orbitals at the Fermi level. Comparison with ambient-pressure bulk samples indicates hole-doping in films, likely due to interfacial Sr diffusion. Complementing this, Yue *et al.* employ density functional theory plus cluster dynamical mean-field theory, incorporating the on-site Coulomb repulsive *U* and experimentally measured electron filling *n*, to successfully reproduce the thin-film electronic band structure [10]. Their analysis suggests an *s*^±^-wave pairing instability driven by strong spin fluctuations and underscores the importance of electronic correlations.

Collectively, these articles highlight significant progress and ongoing challenges in nickelate superconductivity, deepening our understanding of high-*T*_c_ mechanisms. We hope this Special Topic inspires further innovative research in this rapidly evolving field.
